# Surgical Treatment of Iatrogenic Steroid Injection-induced Myelomalacia

**DOI:** 10.1097/GOX.0000000000002026

**Published:** 2018-12-12

**Authors:** Matthew R. Kaufman, Eric I. Chang, Kristie Rossi, Zuhaib Ibrahim, Andrew I. Elkwood

**Affiliations:** From the The Institute for Advanced Reconstruction, The Plastic Surgery Center, Shrewsbury, N.J.

Treatment of cervical radiculopathy often involves conservative measures including steroid injections.^1–3^ However, these procedures do carry associated risks.^4^ Here, we present a case of a 49-year-old woman who experienced immediate respiratory compromise and paralysis due to inadvertent intramedullary injection of steroid. Although she regained some degree of motor function of the left upper and lower extremities, there was decreased strength with pronation, supination, and wrist and finger extension with significant wasting of the intrinsic muscles on the right side. Furthermore, she also experienced persistent loss of sensation along the distribution of the right tibial and medial and lateral plantar nerves and the right antebrachial cutaneous nerve.

**Fig. 1.**
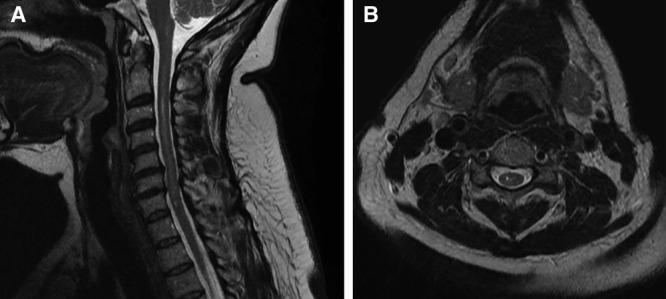
Cervical spine magnetic resonance imaging T2-weighted sagittal (A) and axial (B) images demonstrating degenerative changes and myelomalacia extending to the right dorsal medulla and cervical cord.

Serial radiographic imaging showed intramedullary contrast extending from the occiput to C7 and extension into the medulla. A magnetic resonance imaging performed 14 months later demonstrated degenerative changes and myelomalacia extending to the right dorsal medulla and cervical cord (Fig. 1). An electromyogram (EMG) suggested multilevel root dysfunction in the distribution of C5-T1 with decreased distal motor and sensory function of the right upper extremity.

The patient presented more than 2 years after the initial injury and underwent exploration and decompression of the right carpal tunnel, cubital tunnel, and Guyon’s canal and the right tarsal tunnel. Microneurolysis and epineurectomy was performed of the ulnar nerve of the arm and elbow and the nerve to the flexor carpi ulnaris. The tibial nerve, medial plantar nerve, and lateral plantar nerve were also serially released. The sural nerve was harvested from the bilateral lower extremities and used as grafts from the left greater auricular nerve and the left supraclavicular nerve to the medial cutaneous nerve of the right arm and forearm in an end-to-side fashion respectively. The medial cutaneous nerve of the arm was also neurotized into a partial neurotomy of the sensory component of the ulnar nerve.

There were no postoperative complications and the patient reported improved sensation in the right hand and forearm after 4 weeks and increased grip strength accompanied by a positive Tinel sign at 10 weeks. Sensation in the right upper extremity continued to improve with an advancing Tinel sign across the chest, and the patient reported significant improvement in the sensation of the sole of foot. After 13 months, sensation returned to the tips of the fingers with 6–7 mm 2-point discrimination. Even though intrinsic hand muscle weakness was still apparent, there was improved function of the flexi carpal ulnaris and the fourth and fifth flexor digitorum profundus muscles.

The treatment of cervical radiculopathy is particularly challenging but becomes even more devastating in a young patient, especially when the symptomatology is compounded by myelomalacia due to an iatrogenic injury. Fortunately, exploration and decompression of the right upper extremity nerves at the level of the carpal tunnel, cubital tunnel, and Guyon’s canal with bilateral sural nerve harvest and contralateral grafting was successful. Certainly, evaluation and treatment by a multidisciplinary team with experience in reconstructive surgery with peripheral nerves and the brachial plexus are paramount in achieving optimal outcomes.
